# A Subclone of HuH-7 with Enhanced Intracellular Hepatitis C Virus Production and Evasion of Virus Related-Cell Cycle Arrest

**DOI:** 10.1371/journal.pone.0052697

**Published:** 2012-12-20

**Authors:** Asako Murayama, Nao Sugiyama, Seiko Yoshimura, Mitsuko Ishihara-Sugano, Takahiro Masaki, Sulyi Kim, Takaji Wakita, Shunji Mishiro, Takanobu Kato

**Affiliations:** 1 Department of Virology II, National Institute of Infectious Diseases, Tokyo, Japan; 2 Corporate Research and Development Center, Toshiba Corporation, Kanagawa, Japan; 3 Department of Medical Sciences, Toshiba General Hospital, Tokyo, Japan; University of Tennessee Health Science Center, United States of America

## Abstract

Hepatitis C virus (HCV) cell culture system with JFH-1 strain and HuH-7 cells enabled us to produce infectious HCV particles *in vitro*, and such system is useful to explore the anti-HCV compounds and to develop the vaccine against HCV. In the present study, we describe the derivation of a cell line that permits improved production of HCV particles. Specifically, we characterized several subclones that were isolated from the original HuH-7 cell line by limiting dilution. These HuH-7 subclones displayed a notable range of HCV production levels following transfection by full-genome JFH-1 RNA. Among these subclones, HuH-7T1 produced HCV more efficiently than other subclones and Huh-7.5.1 that is known to be highly permissive for HCV replication. Upon transfection with full-genome RNA, HCV production was increased ten-fold in HuH-7T1 compared to Huh-7.5.1. This increase in viral production correlated with increased efficiency of intracellular infectious virus production. Furthermore, HCV replication did not induce cell cycle arrest in HuH-7T1, whereas it did in Huh-7.5.1. Consequently, the use of HuH-7T1 as host cells could provide increased population of HCV-positive cells and elevated viral titer. In conclusion, we isolated a HuH-7 subclone, HuH-7T1, that supports efficient HCV production. High efficiency of intracellular infectious virus production and evasion of cell cycle arrest were important for this phenotype. We expect that the use of this cell line will facilitate analysis of the underlying mechanisms for HCV particle assembly and the cell cycle arrest caused by HCV.

## Introduction

Hepatitis C virus (HCV) is a major cause of chronic liver disease [1], [2]. Currently, approximately 200 million people are infected with HCV worldwide and are at continued risk of developing chronic liver diseases such as chronic hepatitis, liver cirrhosis, and hepatocellular carcinoma [3], [4]. Historically, the lack of a cell culture system capable of producing virus particles hampered progress in the field of HCV research. Subsequently, a robust HCV cell culture system was developed using HCV JFH-1 strain that had been cloned from a fulminant hepatitis patient [5], [6], [7]. JFH-1 was the first HCV strain that could replicate and produce HCV particles autonomously *in vitro*, thereby facilitating investigation of the entire life cycle of the virus. This HCV cell culture system employed HuH-7 cell line, which was established from a hepatocellular carcinoma [5], [8], as a host. Since the HCV replicon system enabling HCV subgenomic RNA replication was originally developed using HuH-7 [9], this cell line has been used in the research field of HCV most frequently. However, HuH-7 is known to be heterogeneous. Notably, Saintz et al. reported that HuH-7 cell lines obtained from various laboratories exhibit distinct morphological, cell growth, and HCV susceptibility properties [10]. We also found that single-cell cloning of HuH-7 maintained in our laboratory yielded multiple subclones that exhibited different characteristics of HCV infection and replication [11]. In the present study, we derived cell lines from original HuH-7 obtained from the cell bank and screened to identify a cell line with improved production of infectious HCV particles. As we report here, we obtained one such clone (HuH-7T1) and performed an initial characterization of the HCV life cycle in this host.

## Materials and Methods

### Cell culture

The original HuH-7 cell line (catalog number; JCRB0403) was purchased from Health Science Research Resources Bank (Osaka, Japan). The cured cell line, Huh-7.5.1, was a kind gift from Dr. Francis V. Chisari (Scripps Research Institute, La Jolla, CA) [6]. These cell lines were cultured at 37°C in a 5% CO_2_ environment using Dulbecco's Modified Eagle's Medium containing 10% fetal bovine serum.

### Single cell cloning by limiting dilution

The original HuH-7 cell line was diluted with medium at 1 cell/mL and seeded at 100 μL/well in 96-well plates. Six subclones were obtained and resulting subclones were expanded and stored at −80°C pending further characterization. The characteristics of obtained subclones were maintained after passages over several months.

### HCV constructs and RNA transfection

pJFH1 is a full-length JFH-1 clone whose construction was reported previously [5]. pSGR-JFH1-Luc (a JFH-1 subgenomic replicon construct containing a firefly luciferase-encoding reporter gene) and pSGR-JFH1/GND-Luc (a replication-defective mutant construct) also were described previously [12]. pH77S.2, a full-length H77S.2 construct, was a kind gift from Dr. Stanley M Lemon (University of North Carolina at Chapel Hill, Chapel Hill, NC). This construct is a derivative of strain H77S (genotype 1a) harboring an additional mutation, and produces infectious virus in cultured cells after full-genome RNA transfection [13]. RNA synthesis and transfection were performed as described previously [14], [15].

### Quantification of HCV core protein and RNA

The concentration of HCV core protein in the culture medium and cell lysate was measured using a chemiluminescent enzyme immunoassay (Lumipulse Ortho HCV antigen, Fujirebio, Tokyo, Japan) in accordance with the manufacturer's instructions. The concentration of HCV RNA was measured as described previously [16].

### Determination of infectivity titers

To determine the intracellular infectivity of the HCV RNA-transfected cells, a cell lysate of HCV RNA-transfected cells cultured in a 10 cm dish was generated by subjecting the cells to four freeze-thaw cycles. The culture supernatant and cell lysate were serially diluted and inoculated into naive Huh-7.5.1 seeded at 1×10^4^ cells/well in poly-D-lysine-coated 96-well plates (BD, Franklin Lakes, NJ), and the inoculated plates were incubated for another 3 days at 37°C. The cells were then fixed with methanol, and the infected foci were visualized by staining with anti-core antibody (clone 2H9 [5], [8] for JFH-1 and c7-50 (Abcam, Cambridge, MA) for H77S.2) and Alexa Fluor 488 Goat Anti-mouse IgG (Invitrogen, Carlsbad, CA). The infectivity titer was quantified by counting the stained foci and expressing the value as the number of focus-forming units (FFU).

### Flow cytometric analysis

For cell cycle distribution analyses, cells were labeled with 5-ethynyl-2′-deoxyuridine (EdU) for 4 h prior to harvest. The harvested cells were fixed in 4% paraformaldehyde, permeabilized, and stained with anti-nonstructural (NS) 5A antibody (clone KS0265-1; raised by immunization with JFH-1 NS5A) and Alexa Fluor 647 Goat Anti-mouse IgG (Invitrogen). Incorporated EdU was stained with Alexa Fluor 488 azide by using the Click-iT EdU flow cytometry kit (Invitrogen) according to the manufacturer's instructions. Following treatment with RNase A, 7-aminoactinomycin D (7-AAD) was added. Samples were analyzed using a FACS Calibur flow cytometer. The population of cells in G0/G1, S, or G2/M phases of the cell cycle was determined using FlowJo software (Tree Star, Inc., Ashland, OR).

### Immunostaining

Infected cells were cultured on glass cover slips in a 12-well plate. Cells were fixed in 4% paraformaldehyde and permeabilized. After blocking, HCV-positive cells were visualized by staining with anti-core antibody (clone 2H9) and Alexa Fluor 488 Goat Anti-mouse IgG, and nuclei were stained with 4′, 6-diamidino-2-phenylindole (DAPI).

### Virus entry assay

HCV pseudo type virus (HCVpp) harboring the JFH-1 E1 and E2 glycoprotein was prepared as described previously [11]. Target cells were seeded into 48-well plates at a density of 2×10^4^ cells/well. On the following day, a 100-μL aliquot of each diluted supernatant containing HCVpp was added to each well and incubated for 3 h. The supernatants were replaced with fresh medium, and the cells were incubated for 72 h at 37°C. Cells were lysed with Passive Lysis Buffer (Promega, Madison, WI). Luciferase activities were quantified using a luciferase assay system (Promega). Assays were performed in triplicate; data are presented as mean ± standard deviation.

Cell culture-generated HCV JFH-1 virus (HCVcc) was prepared as follows: culture medium from JFH-1 RNA-transfected cells was collected and 40-times concentrated using Amicon Ultra-15 filter units (100-kDa cutoff; Millipore, Bedford, MA) and stored at −80°C until use. HCVcc was inoculated into target cells, and infectivity titer was determined as described.

### Luciferase assay

Luciferase activity of subgenomic reporter replicon RNA-transfected cell lysate was measured as described previously [14], [15].

### Statistical analysis

Significant differences were evaluated using the Student's t-test. *P*<0.05 was considered significant.

## Results

### Isolation of HuH-7 subclones with improved HCV production

**Figure 1:**
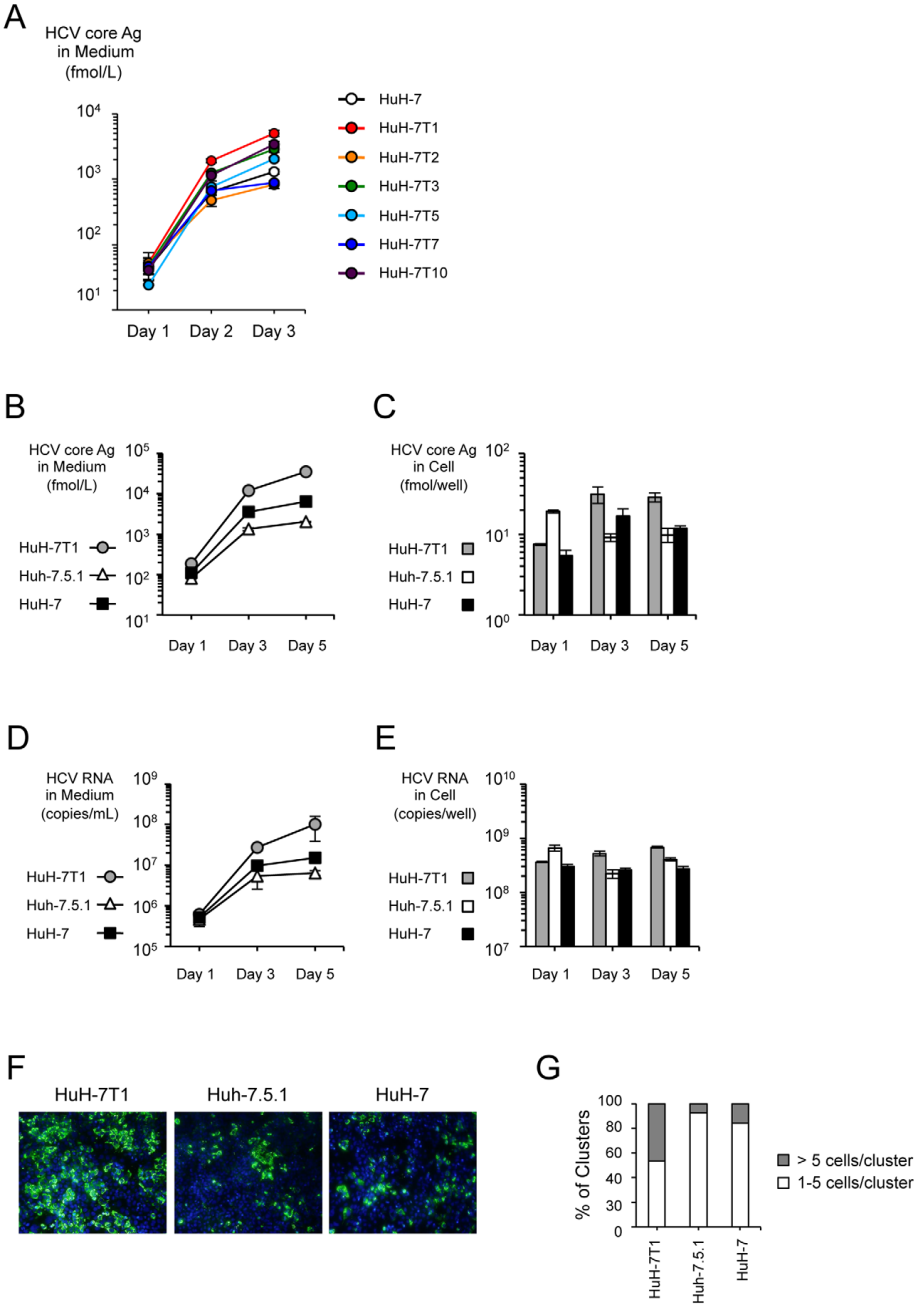
HCV production in HuH-7 subclones. (A) Two micrograms of JFH-1 RNA were electroporated into the HuH-7 subclones. Culture medium was harvested at Days 1, 3, and 5, and HCV core protein levels in the culture medium were measured. Assays were performed three times independently, and data are presented as mean ± standard deviation. (B–D) Comparison of HCV production among HuH-7T1, Huh-7.5.1 and HuH-7. HCV core protein (B and C) and HCV RNA (D and E) levels in cells and culture medium were measured. Assays were performed three times independently, and data are presented as mean ± standard deviation. (F) HCV-positive cells at Day 3 post-transfection were visualized with anti-core antibody (green); nuclei were visualized with DAPI (blue). (G) The number of HCV positive cells within a cluster were counted and classified into 2 groups (>5 cells/cluster and 1–5/cluster). More than 100 foci were counted. The percentages of each group are shown.

To obtain cell lines with improved HCV production potential, we used limiting dilution to establish six subclones (HuH-7T1, HuH-7T2, HuH-7T3, HuH-7T5, HuH-7T7, and HuH-7T10) from the original HuH-7 purchased from the cell bank. We transfected JFH-1 RNA into each of these subclones and measured the level of core protein in the culture medium. These subclones displayed a range of core protein production levels. (Fig. 1A). Compared to the original HuH-7, four (HuH-7T1, HuH-7T3, HuH-7T5 and HuH-7T10) and two (HuH-7T2 and HuH-7T7) subclones produced higher or lower amounts of HCV core protein, respectively. Among these subclones, we chose HuH-7T1 for further characterization because this subclone produced HCV core protein at the highest level (Fig. 1A). Then, we compared core protein production of HuH-7T1 with Huh-7.5.1, a cell line reported to be highly permissive for HCV replication [6]. After JFH-1 RNA transfection, HCV core protein level in the culture medium of HuH-7T1 was 17.6-fold higher than that seen with Huh-7.5.1 (Fig. 1B). HCV core protein levels in cell lysate of HuH-7T1 were lower at Day 1, but higher at Days 3 and 5 after transfection, compared to Huh-7.5.1 (Fig. 1C). HCV RNA levels in the culture medium and cell lysates of these cells showed similar tendencies (Fig. 1D and 1E). The infectivity titer in culture medium of HuH-7T1 at Day 5 was 22.5-fold higher than that of Huh-7.5.1 (Table 1), indicating that HuH-7T1 supported production of infectious HCV particles to levels higher than those seen in Huh-7.5.1. The number of HCV-positive cells of HuH-7T1 at Day 5 also was higher than that seen with Huh-7.5.1 (Fig. 1F). The percentage of HCV positive cell clusters consisting of more than 5 cells was higher in HuH-7T1 than in Huh-7.5.1 (Fig. 1G). We also assessed if HuH-7T1 produced higher amount of core protein after infection of HCVcc. HuH-7T1 produced higher amount of HCV core protein than Huh-7.5.1 after JFH-1 virus infection at the same multiplicity of infection ( Fig. S1A), and HCV core protein levels in cell lysate of HuH-7T1 were also higher than that of Huh-7.5.1 (Fig. S1B). These data indicated that HuH-7T1 produced infectious HCV particles more efficiently than Huh-7.5.1 after JFH-1 RNA transfection and JFH-1 virus infection.

**Table 1:** Infectivity titers in culture medium and cells of HuH-7T1 and Huh-7.5.1 transfected with JFH-1 RNA.

Cell Line	Infectivity		Secretion Rate
	Medium (FFU/dish)	Cells (FFU/dish)	
HuH-7T1	2.23×10^6^±3.15×10^5^ *	1.11×10^4^±1.15×10^3^ *	2.00×10^2^±1.98×10^1^ *
Huh-7.5.1	9.92×10^4^±2.98×10^4^	1.34×10^2^±1.42×10^1^	7.30×10^2^±1.40×10^2^

* *P*<0.05 as compared with Huh-7.5.1.

The original HuH-7 could produce higher amount of HCV core protein than Huh-7.5.1 after JFH-1 RNA transfection (Fig. 1B). However, in the experiment of HCVcc infection, HuH-7 produced lower amount of HCV core protein than Huh-7.5.1 in culture medium (Fig. S1A) and in cell lysate (Fig. S1B).

### Analysis of HCV life cycle in HuH-7T1

**Figure 2:**
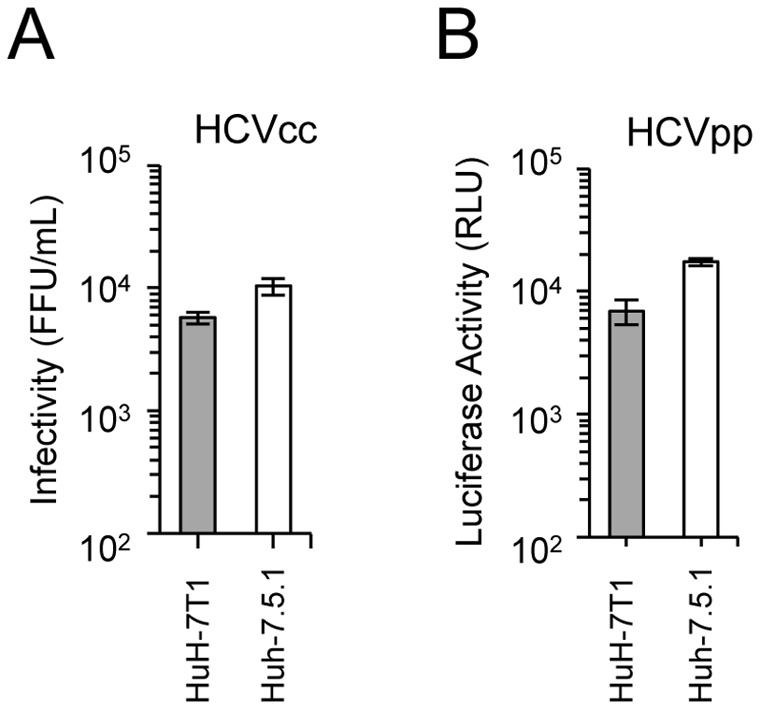
Comparison of infection in HuH-7T1 and Huh-7.5.1. (A) Infection of HCVcc into HuH-7T1 and Huh-7.5.1. The cells were fixed 3 days after infection and infected foci were counted. (B) Infection of HCVpp into HuH-7T1 and Huh-7.5.1. The cells were harvested 3 days after infection, and the luciferase activity in the cell lysate was measured.

To clarify the underlying mechanism of the enhanced virus production in HuH-7T1, we assessed the efficiencies of each step in the HCV life cycle. The viral infection step was assessed by using HCVcc and HCVpp. The HCVcc system uses cell culture-generated HCV and detects steps from viral attachment through replication. On the other hand, the HCVpp system uses the retroviral particles harboring the HCV envelope protein and a luciferase reporter gene, and measures infection efficiency in the absence of HCV replication [11]. The infectivity titer of HCVcc in HuH-7T1 was 33.0%±8.1% of that in Huh-7.5.1 (Fig. 2A). To evaluate the infection efficiency of HCVpp, cellular luciferase activity was measured after HCVpp infection. The luciferase activity in HuH-7T1 was 39.5%±9.0% of that in Huh-7.5.1 (Fig. 2B). As there were differences in infection efficiencies of HCVcc and HCVpp between these cell lines, we analyzed cell-surface expression of the HCV receptor, CD81, using flow cytometry. The population of CD81-expressing cells was slightly lower in HuH-7T1 than in Huh-7.5.1, and HuH-7T1 showed a broad peak of CD81 expression, indicating that CD81 expression level in each cell varied (Fig. S2). Taken together, these results indicated that the susceptibility for HCV infection in HuH-7T1 was lower than in Huh-7.5.1. This distinction presumably reflected the reduced population of CD81-expressing cells, implying that this step was not responsible for the enhanced virus production in HuH-7T1.

**Figure 3:**
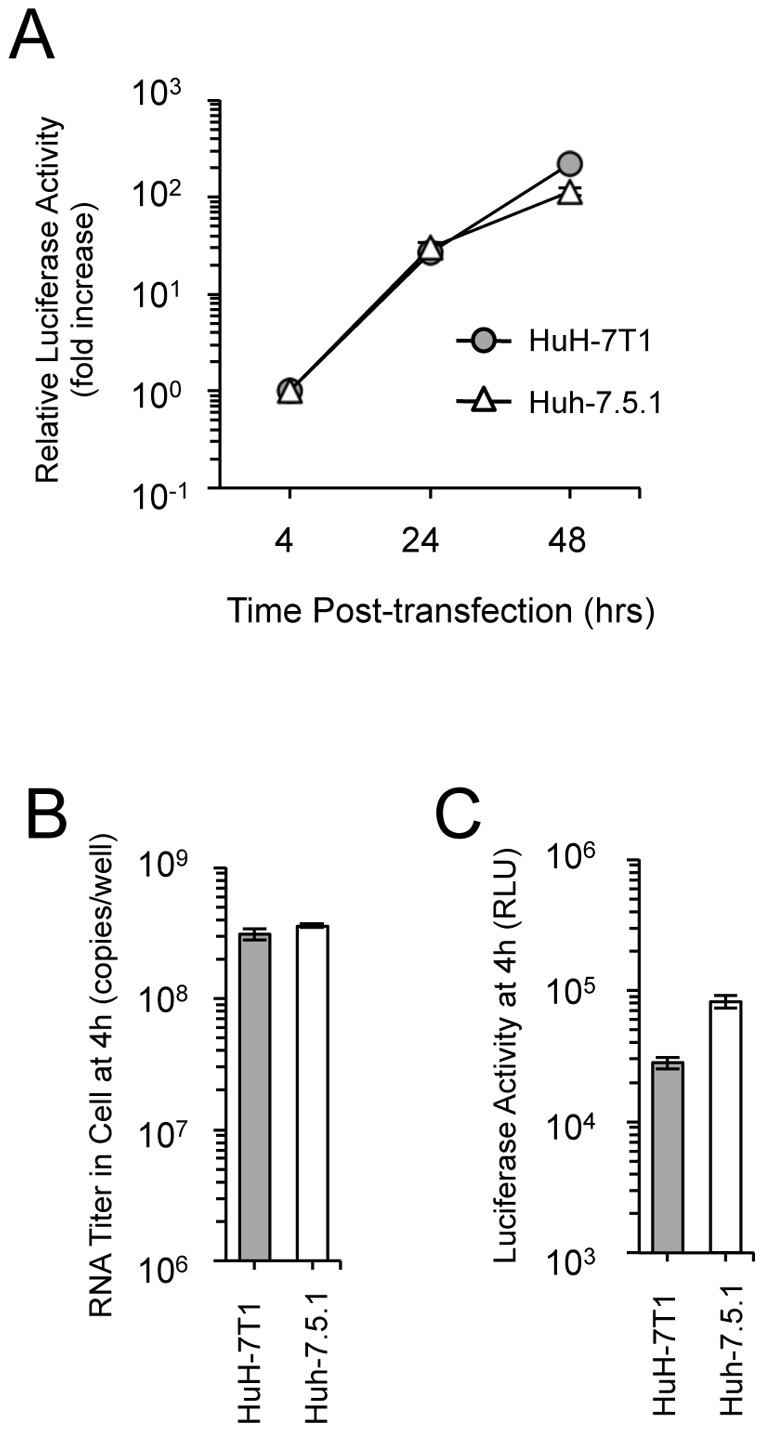
Comparison of replication in HuH-7T1 and Huh-7.5.1. (A) Five micrograms of JFH-1 subgenomic replicon RNA was electroporated into HuH-7T1 and Huh-7.5.1. The cells were harvested at indicated time points. The luciferase activity in the cell lysates was normalized to the data at 4 h after transfection; values are expressed as fold increases. (B and C) Comparison of transfection and translation efficiencies. Five micrograms of JFH-1/GND-Luc RNA was transfected into HuH-7T1 and Huh-7.5.1. The cells were harvested at 4 h after transfection, and the amount of transfected RNA in cells (B) and luciferase activity in the cell lysates (C) were measured.

We assessed RNA replication efficiency by transfection with a subgenomic JFH-1 replicon RNA that harbored a luciferase-encoding gene. Subgenomic replicon assay revealed that RNA replication in HuH-7T1 demonstrated similar kinetics to that seen in Huh-7.5.1 when compared with the fold-increase value over 4 h of each cells (Fig. 3A), but the absolute luciferase activities of HuH-7T1 were lower than that of Huh-7.5.1 at all time points tested ( Fig. S3). We then compared RNA transfection efficiency by measuring the RNA titers of the transfected replication-defective subgenomic replicon RNA (SGR-JFH1/GND-Luc) in the cells. The amount of replicon RNA in the two cell lines was same level at 4 h after transfection (Fig. 3B). However, the luciferase activity in HuH-7T1 was 2.9-times lower than that of Huh-7.5.1 at 4 h after transfection (Fig. 3C). Thus, translation efficiency of HCV genome was lower in HuH-7T1 than in Huh-7.5.1. Taken together, neither the translation or replication step was responsible for enhanced virus production in HuH-7T1.

To assess the efficiencies of intracellular infectious virus production and secretion, we compared infectivity titers in cells and medium of JFH-1 RNA-transfected HuH-7T1 and Huh-7.5.1. At Day 5 after transfection, the intracellular infectivity of HuH-7T1 was 83-fold higher than that of Huh-7.5.1 (Table 1). However, the core protein level of the cells of HCV RNA-transfected HuH-7T1 at Day 5 was only 2.9-fold higher than that of Huh-7.5.1 (Fig. 1B), indicating that infectious HCV particles were assembled more efficiently in HuH-7T1 than in Huh-7.5.1. Virus secretion efficiencies also were assessed by comparing the ratio of infectivity titers in cells and supernatants, and were 3.7-fold lower in HuH-7T1 compared to Huh-7.5.1 (Table 1). Taken together, these results indicated that the efficiency of intracellular infectious virus production was significantly higher in HuH-7T1 than that in Huh-7.5.1, whereas virus secretion efficiency was slightly lower in HuH-7T1 than that in Huh-7.5.1. Therefore, the enhanced intracellular infectious virus production was considered to be responsible for the advantage of HuH-7T1.

### Gene expression analysis

To identify the host factors regarding the concerned properties of HuH-7T1, we measured gene expression levels for genes that encode cellular factors reported to be involved in the HCV life cycle. Among 37 host factor-encoding transcripts tested, none except for miR-122 showed more than 2-fold higher or lower expression levels in HuH-7T1 compared to Huh-7.5.1 (Fig. S4). The gene expression level of miR-122 was approximately 5-times lower in HuH-7T1 than in Huh-7.5.1.

### Cell cycle analysis of HuH-7T1 and Huh-7.5.1

**Figure 4:**
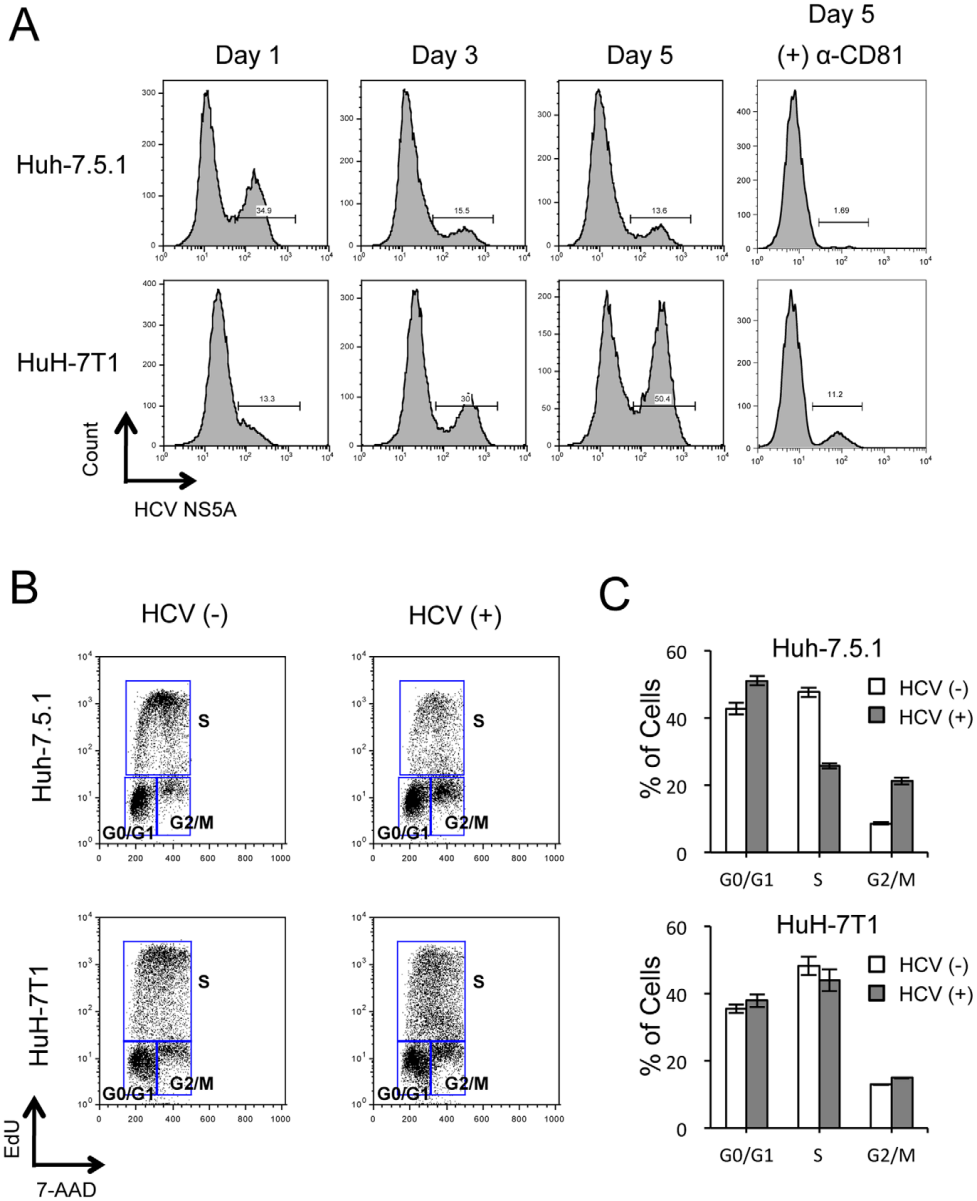
Effects of HCV replication on cell proliferation of Huh-7.5.1 and HuH-7T1. (A) Population of HCV-positive cells after JFH-1 RNA transfection. Two micrograms of JFH-1 RNA was electroporated into Huh-7.5.1 and HuH-7T1 and cultured with or without 10 mg/mL of anti-CD81 antibody (clone JS-81, BD). Cells were harvested at Days 1, 3, and 5. After fixing, cells were stained with anti-NS5A antibody and analyzed by flow cytometry. (B, C) Cell cycle distribution of HCV-positive and -negative cells after JFH-1 RNA transfection. Two micrograms of JFH-1 RNA was electroporated into Huh-7.5.1 and HuH-7T1. Cells were pulse-labeled with EdU and analyzed for cell cycle distribution. The percentages of cells in G0/G1, S, and G2/M phases of the cell cycle were calculated by gating with FlowJo software. (B) Representative cell cycle distributions of HCV-negative and -positive cells. (C) Percentages of cells in each phase of the cell cycle for HCV-negative and -positive populations. Assays were performed three times independently; data are presented as mean ± standard deviation.

**Figure 5:**
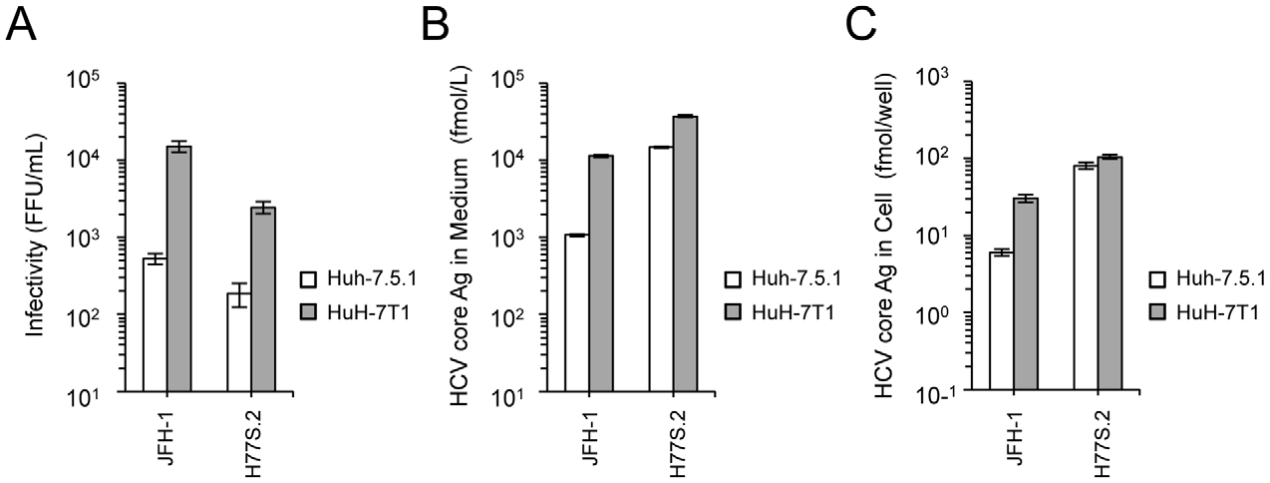
Infectious virus production of H77S.2 in HuH-7T1 and Huh-7.5.1. Two micrograms of JFH-1 RNA or 10 micrograms of H77S.2 RNA were electroporated into Huh-7.5.1 and HuH-7T1. Culture medium and cells were harvested at Day 3, and infectivity titer and HCV core level were determined.

Although we found that intracellular infectious HCV particles produced more efficiently in HuH-7T1 than in Huh-7.5.1, we thought that there were other possible steps associated with the efficient virus production of HuH-7T1. Because, when HCV RNA is transfected, HuH-7T1 forms the larger HCV positive cell clusters than Huh-7.5.1 (Fig. 1G), although viral entry is less efficient in HuH-7T1 as compared with Huh-7.5.1. To determine other advantages of HuH-7T1, we used flow cytometry to monitor the population of the HCV-positive cells after RNA transfection. At Day 1, the population of HCV-positive cells was higher in Huh-7.5.1 (34.9%) than in HuH-7T1 (13.3%) (Fig. 4A). However, the population of HCV-positive cells in HuH-7T1 increased from Day 1 to Day 5, while that in Huh-7.5.1 decreased over the same interval. When we added anti-CD81 antibody to the medium to exclude the effect of re-infection of the progeny virus, we found that the population of HCV-positive cells in HuH-7T1 did not change from Day 1 to Day 5, while that in Huh-7.5.1 decreased more severely. From these data, we hypothesized that proliferation of HCV-positive cells differed between these cell lines. To clarify this point, we compared the cell cycle distribution of HCV-positive and -negative cells after JFH-1 RNA transfection (Fig. 4B). In Huh-7.5.1, the fraction of cells in S phase was lower among HCV-positive cells than among HCV-negative cells (25.7%±0.8% vs 47.6%±1.5%, respectively; *P*<0.05; Fig. 5C); conversely, the fraction of cells in G0/G1 and G2/M phases was higher among HCV-positive cells compared to HCV-negative cells (51.0%±1.4% vs. 42.8%±1.7%, 21.2%±1.1% vs. 8.6%±0.4%, respectively; *P*<0.05, Fig. 4C), indicating that cell proliferation was suppressed by HCV replication in Huh-7.5.1. By contrast, in HuH-7T1, the fraction of cells in S phase was not significantly different for HCV-positive and -negative cells (Fig. 4C). Thus, unlike Huh-7.5.1, HuH-7T1 evaded the cell cycle arrest associated with HCV replication.

We also analyzed HCV-related apoptosis by terminal deoxynucleotidyl transferase-mediated deoxyuridine triphosphate nick-end labeling (TUNEL) assay and found that apoptosis was observed in a limited number of HCV-positive cells (Fig. S5) as we reported previously [17].

### Comparisons of virus production level of H77S.2 (genotype 1a) between HuH-7T1 and Huh-7.5.1

To test whether HuH-7T1 could enhance viral production of HCV strains other than JFH-1, we transfected H77S.2 RNA into HuH-7T1 and Huh-7.5.1 and compared the infectious virus production. As seen with JFH-1 RNA transfection, H77S.2 RNA transfection of HuH-7T1 resulted in increased (13.1-fold) levels of infectious virus and increased (2.5-fold) level of HCV core in medium compared to Huh-7.5.1 (Fig. 5A and 5B), although intracellular HCV core was slightly higher in HuH-7T1 than in Huh-7.5.1 (Fig. 5C).

## Discussion

Increased efficiency of virus production can be achieved by viral adaptations associated with enhancement of steps in the viral life cycle. A number of adaptive mutations that could enhance viral genome replication or viral particle assembly has been reported, although the effects of some of these mutations were strain specific [18], [19], and none of these has been reported to be applicable to multiple strains and genotypes. Therefore, to obtain the efficient virus production with multiple HCV strains, several cell lines permissive for HCV have been established [6], [20], [21], [22], [23]. Generally, they were generated by curing replicon cells in which HCV subgenomic replicon replicated efficiently. As a result, these cured cells support primarily the HCV RNA replication and it is not sufficient to obtain large amounts of virus. The Huh-7.5.1 strain is an example of such a cured cell, and is known to have a loss-of-function mutation in the gene encoding RIG-I, thereby impairing a part of innate immune system and permitting increased HCV replication [20], [24]. In the present study, we used another strategy to obtain the cell line for efficient HCV production, namely the use of limiting dilution to isolate a cell line with the desired properties. Our resulting cell line (designated HuH-7T1) produced infectious virus more efficiently than Huh-7.5.1, while supporting a more rapid increase of HCV infected cells.

To identify the affected steps of the viral life cycle in HuH-7T1, we systematically used various assays to investigate the steps of viral infection, translation, replication, infectious viral particle production, and secretion. The HCV infection step was assessed by two assays, using HCVcc and HCVpp. Both assays indicated that the HCV infection efficiency was lower in HuH-7T1 than in Huh-7.5.1. It has been reported that the susceptibility for HCV infection was associated with CD81 expression levels [11], [25]. We observed that the population of CD81-expressing cells was lower in HuH-7T1 than in Huh-7.5.1. Therefore, the lower infection efficiency of HuH-7T1 was probably due to the reduced number of CD81-expressing cells. We found that the efficiency of genome translation was lower, but the efficiency of replication was similar in HuH-7T1 compared with Huh-7.5.1. By the gene expression analysis, miR-122 was detected as less expressed in HuH-7T1, and it may be responsible for the lower translation efficiency of HuH-7T1. In contrast, the efficiency of intracellular infectious viral particle production was substantially higher in HuH-7T1 than in Huh-7.5.1. We measured the expression levels of genes encoding host factors involved in viral particle assembly, but did not identify any responsible genes for HuH-7T1 phenotype. A comprehensive microarray analysis would be needed to determine the responsible host factors. We also found that virus secretion efficiency was lower in HuH-7T1 than in Huh-7.5.1. Nevertheless, virus production in HuH-7T1 was significantly higher than that in Huh-7.5.1, suggesting that the enhancement of intracellular viral particle production efficiency in HuH-7T1 was sufficient to overcome other disadvantages compared to Huh-7.5.1.

Immunostaining analysis clearly indicated that the number of HCV-positive cells at Day 5 after RNA transfection was larger for HuH-7T1 than for Huh-7.5.1, and the percentage of HCV positive cell clusters consisting of more than 5 cells was higher in HuH-7T1 than in Huh-7.5.1. These effects may not be fully explained by the difference in intracellular viral particle production efficiency. Thus, we focused on the cell proliferation of HCV-replicating cells in HuH-7T1 and Huh-7.5.1. Flow cytometry analysis revealed that the HCV-positive cell population increased in HuH-7T1 from Day 1 to Day 5, in contrast to the decrease seen in Huh-7.5.1 cells during the same interval. A detailed analysis of the cell cycle populations revealed that the ratio of S-phase cells was reduced by HCV replication in Huh-7.5.1, but not in HuH-7T1. Thus, cell proliferation was suppressed by HCV replication in Huh-7.5.1, but not in HuH-7T1. The time-dependent reduction of the HCV-positive cell population observed in Huh-7.5.1 probably resulted from decreased proliferation activity of HCV-replicating cells relative to HCV-negative cells in spite of the efficient re-infection of the progeny virus. In the case of HuH-7T1, the HCV-positive cells could proliferate as like as the HCV-negative cells, and as a result, the HCV-positive cell population was increased by the re-infection of the progeny virus allowing production of large amounts of viruses.

Cell cycle arrest associated with HCV replication in cell culture has been reported previously. Walters et al. observed S-phase reduction in Huh7.5 cells infected with J6/JFH-1 chimeric viruses, but could not identify the factor(s) responsible for the delay in cell cycle progression [26], [27]. Another group also reported an increase in G2/M phase and reduction in S phase in Huh7.5 cells following transfection of JFH-1 and its chimeric viral RNA, and suggested that the degree of cell cycle arrest was related to the intracellular level of viral protein [26], [27]. Additionally, there are numerous papers reporting the relationship between cell cycle arrest and individual HCV proteins such as core [28], [29], [30], NS2 [31] and NS5B [32], [33], [34]. However, effects of these HCV proteins on cell cycle remain controversial, and the mechanisms of cell cycle arrest caused by HCV replication remain unclear. Since HuH-7T1 are resistant to cell cycle arrest by HCV replication while Huh-7.5.1 are sensitive, these cell lines should help to clarify the mechanism of cell cycle arrest while facilitating the identification of host and viral factors involved therein.

The improved viral production in the HuH-7T1 was observed also with another HCV strain, H77S.2. This viral strain is a derivative of H77S [35], which is genotype 1a and produces infectious virus in cultured cells following full-genome RNA transfection [13]. Although the H77S.2 strain could replicate, and secreted HCV core protein more efficiently than JFH-1 in Huh-7.5.1, infectious virus production was less efficient as compared with JFH-1 and infectivity in the medium of H77S.2 RNA-transfected Huh-7.5.1 was at a detectable level. These data implied that H77S.2 mainly secreted unassembled HCV core proteins or noninfectious virus particles. In HuH-7T1, the infectious virus production of H77S.2 was enhanced about ten times, and HCV core level in the medium was enhanced about three times, indicating that HuH-7T1 enhanced infectious virus production. These data also indicated that large amounts of infectious viruses could also be obtained with other HCV strains in HuH-7T1.

In conclusion, we isolated a HuH-7 subclone, HuH-7T1, that displays improved ability to produce infectious HCV virus particles. Enhanced intracellular infectious virus production and evasion of cell cycle arrest were important for the increased efficiency of viral production. This cell line is expected to facilitate HCV research both by providing increased amounts of HCV particles and by permitting the identification of cellular factors involved in viral particle production.

## Supporting Information

Figure S1 **Kinetics of JFH-1 virus infection on HuH-7T1, huh-7.5.1 and HuH-7.** Target cells were seeded into 12-well plates at a density of 2×10^5^ cells/well. On the following day, the cells were infected with JFH-1 virus at a multiplicity of infection of 0.1 and incubated for 72 h at 37°C. Culture medium and cells were harvested at Days 1, 3, and 5, and HCV core protein levels in the culture medium and in the cells were measured. Assays were performed three times independently, and data are presented as mean ± standard deviation.
(TIF)

Figure S2 **Expression levels of CD81 in HuH-7T1 and Huh-7.5.1.** Analysis of CD81 expression on cell surface of HuH-7T1 and Huh-7.5.1 by flow cytometry. The assays were performed three times independently; representative data are shown. Percentages of CD81-positive cells are shown above the histogram.
(TIF)

Figure S3 **Comparison of absolute luciferase activity in HuH-7T1 and Huh-7.5.1.** Absolute measurement data of luciferase activity at Fig. 3A was plotted.
(TIF)

Figure S4 **Expression levels of genes associated with HCV life cycle in HuH-7T1 and Huh-7.5.1.** Total cellular RNA was extracted from Huh-7.5.1 and HuH-7T1, and cDNA was synthesized using Superscript III reverse transcriptase (except for miR-122) or TaqMan MicroRNA RT Kit (miR-122). Quantitative PCR was performed using gene-specific primer and probe sets. Data are expressed as a fold-difference of expression compared to that in Huh-7.5.1. Dashed lines indicate 2-fold higher or lower expression levels compared to Huh-7.5.1.
(TIF)

Figure S5 **Apoptosis assay of JFH-1 RNA-transfected cells.** Two micrograms of JFH-1 RNA was electroporated into Huh-7.5.1 and HuH-7T1. Cells were harvested at Day 3 and fixed in 4% paraformaldehyde, permeabilized, and stained with anti-NS5A antibody (clone KS0265-1) and Alexa Fluor 647 Goat Anti-mouse IgG (Invitrogen). Apoptosis was detected by terminal deoxynucleotidyl transferase-mediated deoxyuridine triphosphate nick-end labeling (TUNEL). Samples were analyzed using a FACS Calibur flow cytometer.
(TIF)

Text S1 **Supporting Materials and Methods.**
(DOC)
