# Supplementary figures and images for: A Subclone of HuH-7 with Enhanced Intracellular Hepatitis C Virus Production and Evasion of Virus Related-Cell Cycle Arrest
Source: PLoS One. 2012 Dec 20;7(12):e52697. doi: 10.1371/journal.pone.0052697 (PMC3527576; doi:10.1371/journal.pone.0052697)

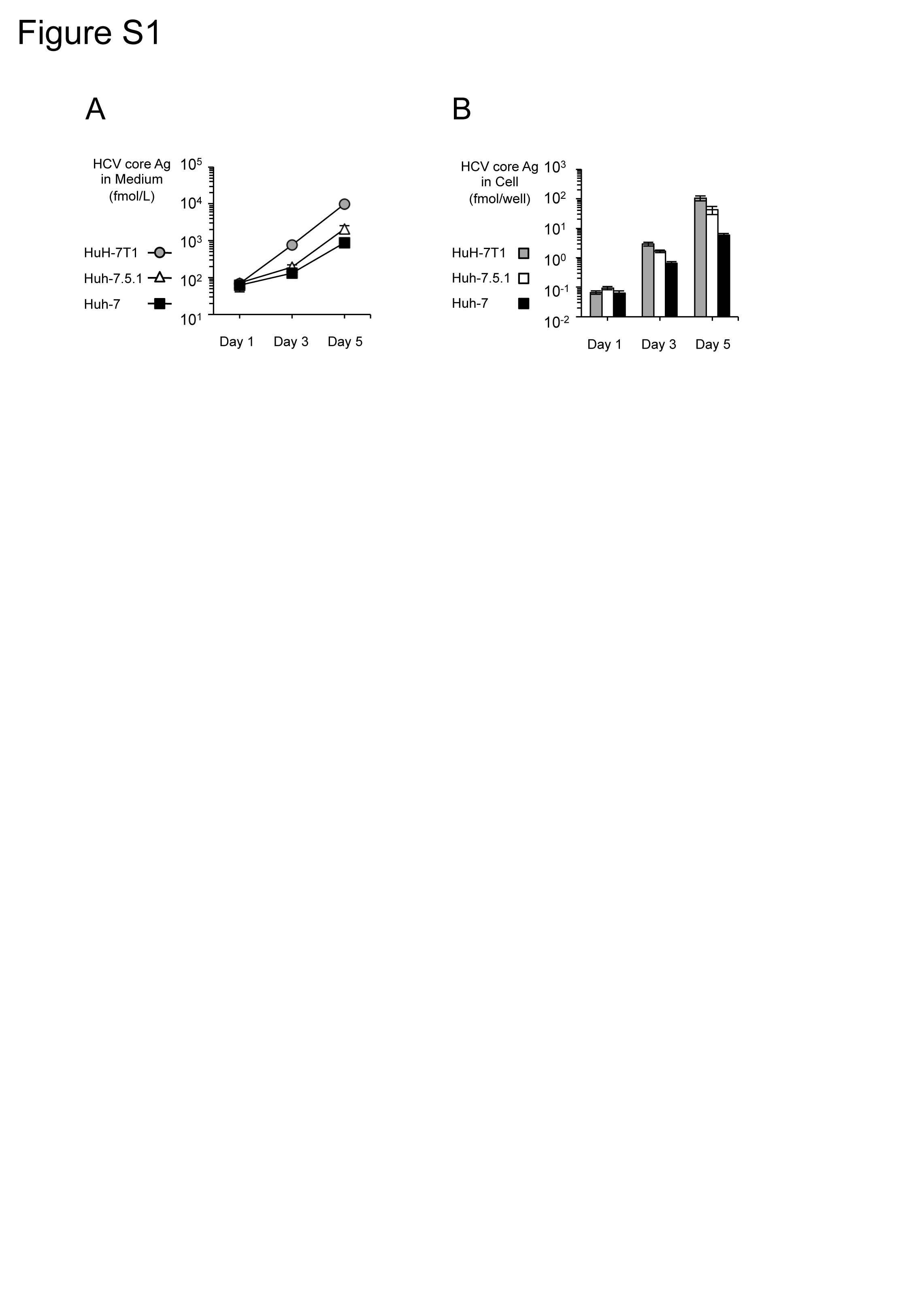

Supplement: Figure S1 — Kinetics of JFH-1 virus infection on HuH-7T1, huh-7.5.1 and HuH-7. Target cells were seeded into 12-well plates at a density of 2×10^5^ cells/well. On the following day, the cells were infected with JFH-1 virus at a multiplicity of infection of 0.1 and incubated for 72 h at 37°C. Culture medium and cells were harvested at Days 1, 3, and 5, and HCV core protein levels in the culture medium and in the cells were measured. Assays were performed three times independently, and data are presented as mean ± standard deviation. (TIF) [file pone.0052697.s001.tif]

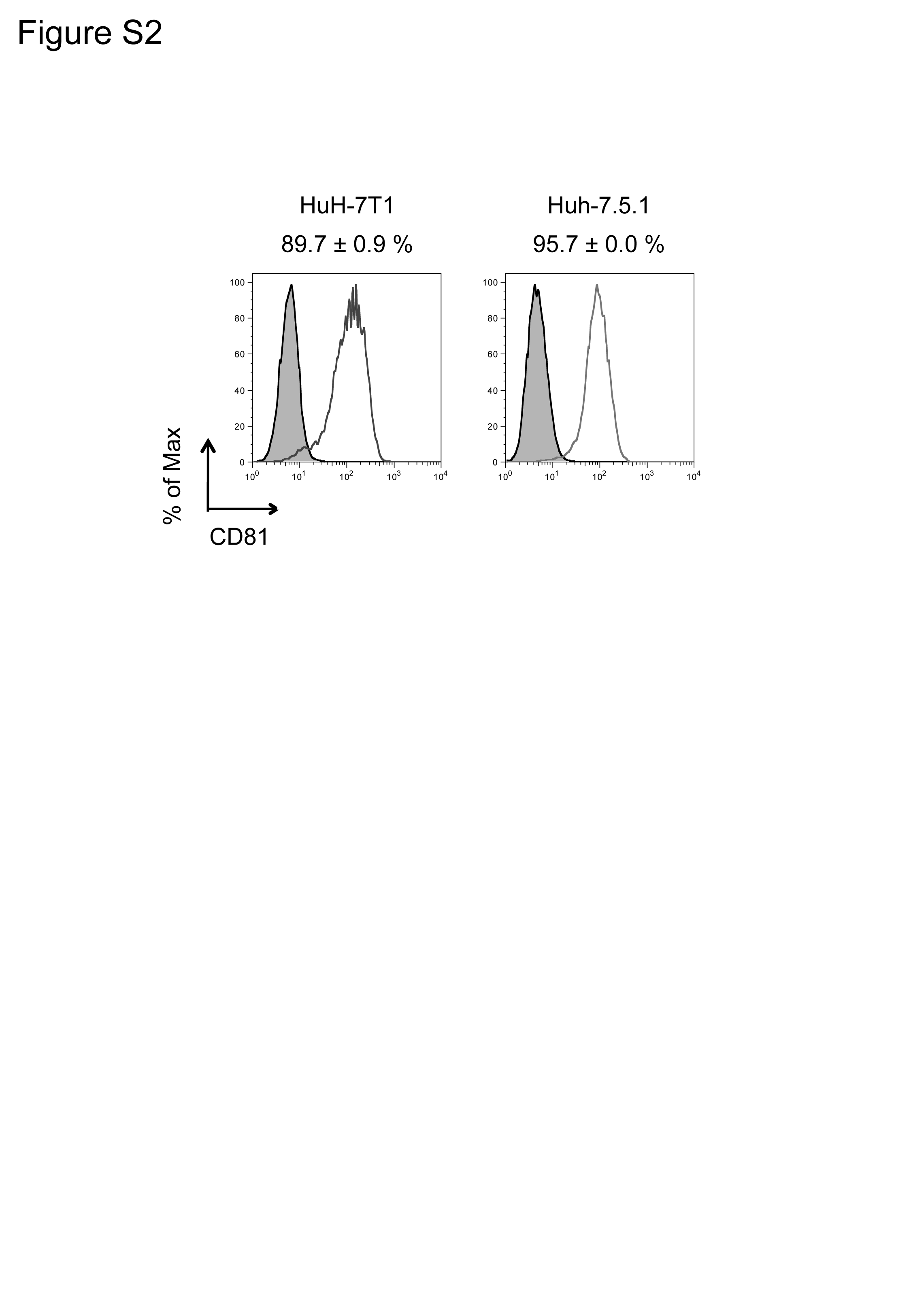

Supplement: Figure S2 — Expression levels of CD81 in HuH-7T1 and Huh-7.5.1. Analysis of CD81 expression on cell surface of HuH-7T1 and Huh-7.5.1 by flow cytometry. The assays were performed three times independently; representative data are shown. Percentages of CD81-positive cells are shown above the histogram. (TIF) [file pone.0052697.s002.tif]

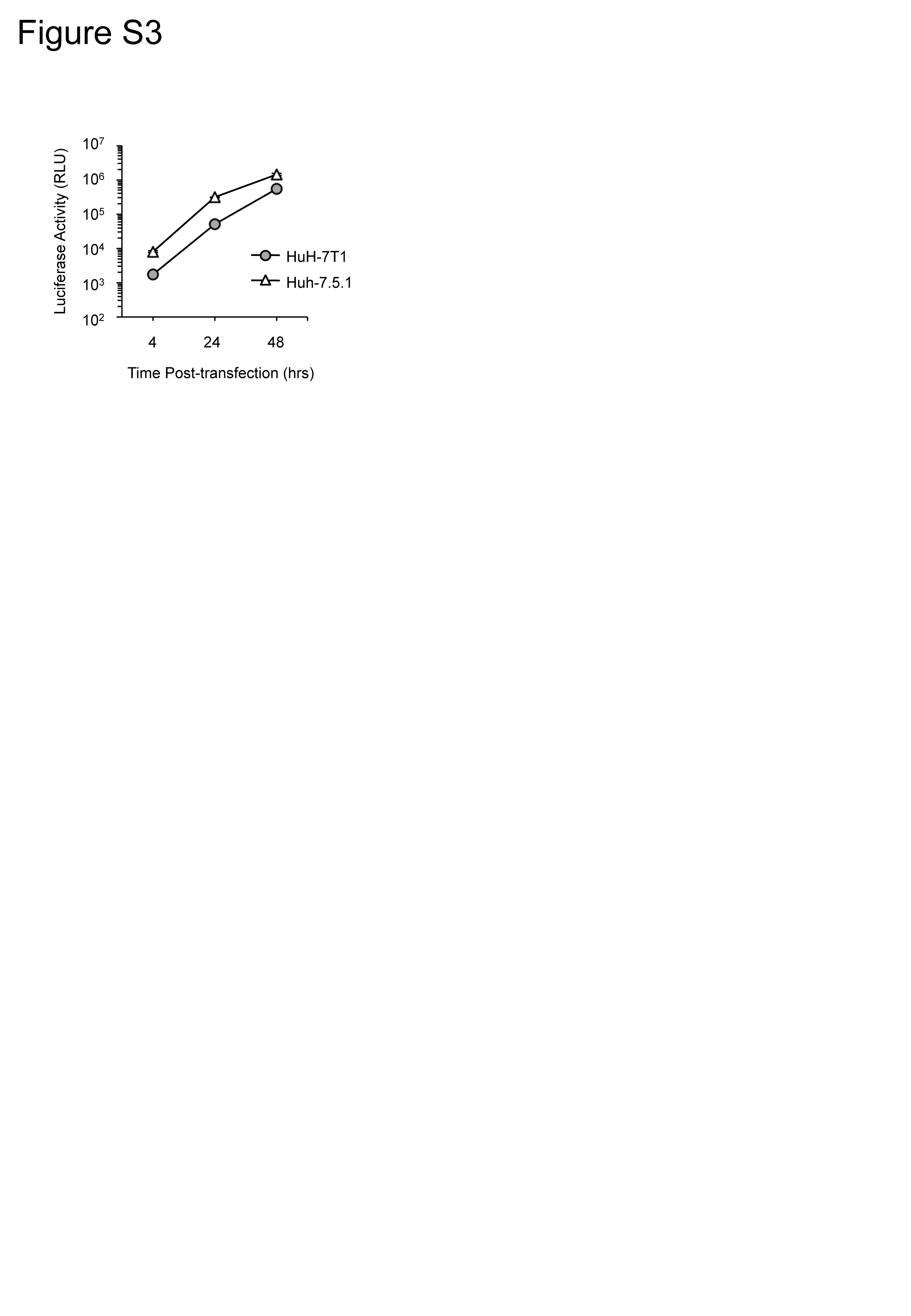

Supplement: Figure S3 — Comparison of absolute luciferase activity in HuH-7T1 and Huh-7.5.1. Absolute measurement data of luciferase activity at Fig. 3A was plotted. (TIF) [file pone.0052697.s003.tif]

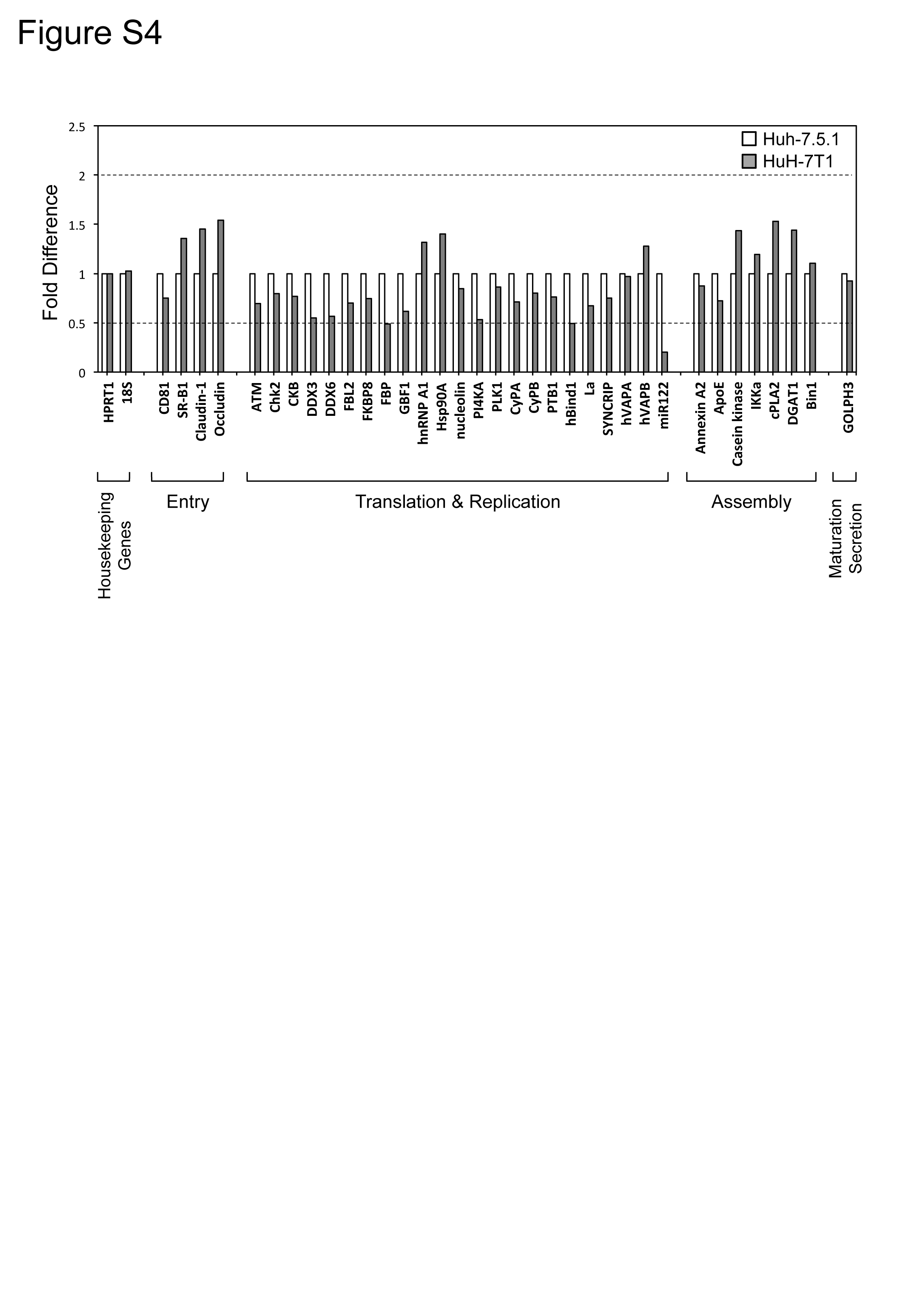

Supplement: Figure S4 — Expression levels of genes associated with HCV life cycle in HuH-7T1 and Huh-7.5.1. Total cellular RNA was extracted from Huh-7.5.1 and HuH-7T1, and cDNA was synthesized using Superscript III reverse transcriptase (except for miR-122) or TaqMan MicroRNA RT Kit (miR-122). Quantitative PCR was performed using gene-specific primer and probe sets. Data are expressed as a fold-difference of expression compared to that in Huh-7.5.1. Dashed lines indicate 2-fold higher or lower expression levels compared to Huh-7.5.1. (TIF) [file pone.0052697.s004.tif]

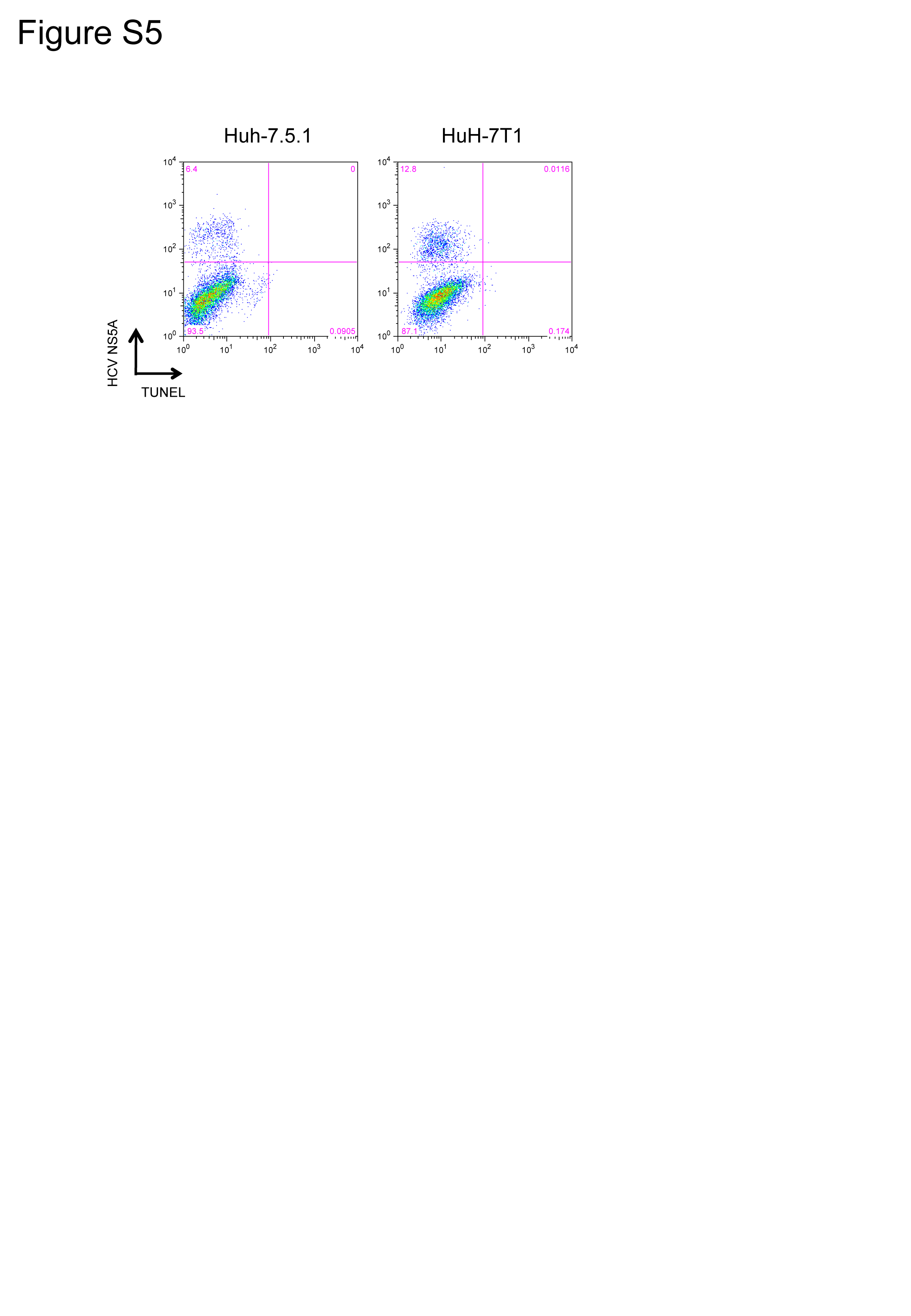

Supplement: Figure S5 — Apoptosis assay of JFH-1 RNA-transfected cells. Two micrograms of JFH-1 RNA was electroporated into Huh-7.5.1 and HuH-7T1. Cells were harvested at Day 3 and fixed in 4% paraformaldehyde, permeabilized, and stained with anti-NS5A antibody (clone KS0265-1) and Alexa Fluor 647 Goat Anti-mouse IgG (Invitrogen). Apoptosis was detected by terminal deoxynucleotidyl transferase-mediated deoxyuridine triphosphate nick-end labeling (TUNEL). Samples were analyzed using a FACS Calibur flow cytometer. (TIF) [file pone.0052697.s005.tif]
