# Supplementary material for: A Subclone of HuH-7 with Enhanced Intracellular Hepatitis C Virus Production and Evasion of Virus Related-Cell Cycle Arrest
Source: PLoS One. 2012 Dec 20;7(12):e52697. doi: 10.1371/journal.pone.0052697 (PMC3527576; doi:10.1371/journal.pone.0052697)
Supplement: Text S1 — Supporting Materials and Methods. (DOC) [file pone.0052697.s006.doc]

**Supporting Materials and Methods**

**JFH-1 infection study**

Target cells were seeded into 12-well plates at a density of 2 × 10^5^ cells/well. On the following day, the cells were infected with JFH-1 virus at a multiplicity of infection of 0.1 and incubated for 4 h. The supernatants were replaced with fresh medium, and the cells were incubated for 72 h at 37°C. The concentration of HCV core protein in the culture medium and cell lysate was measured.

**CD81 expression analysis**

CD81 expressed at the cell surface was detected by staining cells with anti-CD81 antibody (clone JS-81, BD) and Alexa Fluor 488 Goat Anti-mouse IgG, followed by single-cell sorting using a FACS Calibur flow cytometer (Beckman Coulter, Inc., Brea, CA).

**Gene expression analysis**

Total cellular RNA was extracted from Huh-7.5.1 and HuH-7T1 using the RNeasy Mini RNA kit (QIAGEN). cDNA was synthesized from total cellular RNA with random primer (TaKaRa, Shiga, Japan) by using Superscript III reverse transcriptase (Invitrogen). Quantitative PCR was performed using TaqMan Gene Expression Master Mix (Applied Biosystems, Carlsbad, CA) and gene-specific primer and probe sets (TaqMan Gene Expression Assay; Applied Biosystems) in accordance with the manufacturer's instructions. We evaluated expression levels for genes encoding 36 host factors, including 4 infection and entry-related factors (CD81 [1,2,3,4], SR-BI [1,2,5,6], Claudin-1 [7] and Occludin [8,9]), 22 translation and replication-related factors (ATM [10], Chk2 [10], CKB [11], DDX3 [12], DDX6 [13], FBL2 [14], FKBP8 [15,16], FBP [17], GBF1 [18], hnRNP A1 [19], Hsp90A [15], nucleolin [20,21], PI4KA [22], PLK1 [23], CyPA [24], CyPB [25], PTB1 [26,27], hBind1 [28], La [29], SYNCRIP [30], hVAPA [31] and hVAPB [32]), 7 assembly related factors (Annexin A2 [33], ApoE [34], casein kinase II [35], IKKα [36], cPLA2 [37], DGAT1 [38] and Bin1 [39]), and 1 maturation and secretion-related factor (GOLPH3 [40]). Detailed information regarding gene-specific primer and probe sets is available upon request. The expression levels of host factor-encoding genes were normalized to the expression level of the HPRT1-encoding gene in the respective cell line. Data were expressed as the fold-difference of expression level of each host factor-encoding gene relative to that in Huh-7.5.1.

Total cellular RNA including miRNA was extracted from Huh-7.5.1 and HuH-7T1 using the miRNeasy Mini RNA kit (QIAGEN). cDNA of miR-122 and U6 was synthesized from total cellular RNA with gene-specific primer (TaqMan MicroRNA Assay; Applied Biosystems) by using TaqMan MicroRNA RT Kit (Applied Biosystems). Quantitative PCR was performed using TaqMan Universal PCR Master Mix II (Applied Biosystems) and gene-specific primer and probe sets (TaqMan MicroRNA Assay) in accordance with the manufacturer's instructions. The expression level of miR-122 was normalized to the expression level of U6 in the respective cell line. Data were expressed as the fold-difference of expression level of miR-122 relative to that in Huh-7.5.1.

**SUPPLEMENTARY REFERENCES**

1. Bartosch B, Vitelli A, Granier C, Goujon C, Dubuisson J, et al. (2003) Cell entry of hepatitis C virus requires a set of co-receptors that include the CD81 tetraspanin and the SR-B1 scavenger receptor. J Biol Chem 278: 41624-41630.

2. Heo TH, Lee SM, Bartosch B, Cosset FL, Kang CY (2006) Hepatitis C virus E2 links soluble human CD81 and SR-B1 protein. Virus Res 121: 58-64.

3. Hsu M, Zhang J, Flint M, Logvinoff C, Cheng-Mayer C, et al. (2003) Hepatitis C virus glycoproteins mediate pH-dependent cell entry of pseudotyped retroviral particles. Proc Natl Acad Sci U S A 100: 7271-7276.

4. Zhang J, Randall G, Higginbottom A, Monk P, Rice CM, et al. (2004) CD81 is required for hepatitis C virus glycoprotein-mediated viral infection. J Virol 78: 1448-1455.

5. Lavillette D, Tarr AW, Voisset C, Donot P, Bartosch B, et al. (2005) Characterization of host-range and cell entry properties of the major genotypes and subtypes of hepatitis C virus. Hepatology 41: 265-274.

6. Voisset C, Callens N, Blanchard E, Op De Beeck A, Dubuisson J, et al. (2005) High density lipoproteins facilitate hepatitis C virus entry through the scavenger receptor class B type I. J Biol Chem 280: 7793-7799.

7. Evans MJ, von Hahn T, Tscherne DM, Syder AJ, Panis M, et al. (2007) Claudin-1 is a hepatitis C virus co-receptor required for a late step in entry. Nature 446: 801-805.

8. Ploss A, Evans MJ, Gaysinskaya VA, Panis M, You H, et al. (2009) Human occludin is a hepatitis C virus entry factor required for infection of mouse cells. Nature 457: 882-886.

9. Liu S, Yang W, Shen L, Turner JR, Coyne CB, et al. (2009) Tight junction proteins claudin-1 and occludin control hepatitis C virus entry and are downregulated during infection to prevent superinfection. J Virol 83: 2011-2014.

10. Ariumi Y, Kuroki M, Dansako H, Abe K, Ikeda M, et al. (2008) The DNA damage sensors ataxia-telangiectasia mutated kinase and checkpoint kinase 2 are required for hepatitis C virus RNA replication. J Virol 82: 9639-9646.

11. Hara H, Aizaki H, Matsuda M, Shinkai-Ouchi F, Inoue Y, et al. (2009) Involvement of creatine kinase B in hepatitis C virus genome replication through interaction with the viral NS4A protein. J Virol 83: 5137-5147.

12. Angus AG, Dalrymple D, Boulant S, McGivern DR, Clayton RF, et al. (2010) Requirement of cellular DDX3 for hepatitis C virus replication is unrelated to its interaction with the viral core protein. J Gen Virol 91: 122-132.

13. Jangra RK, Yi M, Lemon SM (2010) DDX6 (Rck/p54) is required for efficient hepatitis C virus replication but not for internal ribosome entry site-directed translation. J Virol 84: 6810-6824.

14. Wang C, Gale M, Jr., Keller BC, Huang H, Brown MS, et al. (2005) Identification of FBL2 as a geranylgeranylated cellular protein required for hepatitis C virus RNA replication. Mol Cell 18: 425-434.

15. Okamoto T, Nishimura Y, Ichimura T, Suzuki K, Miyamura T, et al. (2006) Hepatitis C virus RNA replication is regulated by FKBP8 and Hsp90. EMBO J 25: 5015-5025.

16. Okamoto T, Omori H, Kaname Y, Abe T, Nishimura Y, et al. (2008) A single-amino-acid mutation in hepatitis C virus NS5A disrupting FKBP8 interaction impairs viral replication. J Virol 82: 3480-3489.

17. Zhang Z, Harris D, Pandey VN (2008) The FUSE binding protein is a cellular factor required for efficient replication of hepatitis C virus. J Virol 82: 5761-5773.

18. Goueslain L, Alsaleh K, Horellou P, Roingeard P, Descamps V, et al. (2010) Identification of GBF1 as a cellular factor required for hepatitis C virus RNA replication. J Virol 84: 773-787.

19. Kim CS, Seol SK, Song OK, Park JH, Jang SK (2007) An RNA-binding protein, hnRNP A1, and a scaffold protein, septin 6, facilitate hepatitis C virus replication. J Virol 81: 3852-3865.

20. Hirano M, Kaneko S, Yamashita T, Luo H, Qin W, et al. (2003) Direct interaction between nucleolin and hepatitis C virus NS5B. J Biol Chem 278: 5109-5115.

21. Shimakami T, Honda M, Kusakawa T, Murata T, Shimotohno K, et al. (2006) Effect of hepatitis C virus (HCV) NS5B-nucleolin interaction on HCV replication with HCV subgenomic replicon. J Virol 80: 3332-3340.

22. Borawski J, Troke P, Puyang X, Gibaja V, Zhao S, et al. (2009) Class III phosphatidylinositol 4-kinase alpha and beta are novel host factor regulators of hepatitis C virus replication. J Virol 83: 10058-10074.

23. Chen YC, Su WC, Huang JY, Chao TC, Jeng KS, et al. (2010) Polo-like kinase 1 is involved in hepatitis C virus replication by hyperphosphorylating NS5A. J Virol 84: 7983-7993.

24. Kaul A, Stauffer S, Berger C, Pertel T, Schmitt J, et al. (2009) Essential role of cyclophilin A for hepatitis C virus replication and virus production and possible link to polyprotein cleavage kinetics. PLoS Pathog 5: e1000546.

25. Watashi K, Ishii N, Hijikata M, Inoue D, Murata T, et al. (2005) Cyclophilin B is a functional regulator of hepatitis C virus RNA polymerase. Mol Cell 19: 111-122.

26. Ali N, Siddiqui A (1995) Interaction of polypyrimidine tract-binding protein with the 5' noncoding region of the hepatitis C virus RNA genome and its functional requirement in internal initiation of translation. J Virol 69: 6367-6375.

27. Ito T, Lai MM (1997) Determination of the secondary structure of and cellular protein binding to the 3'-untranslated region of the hepatitis C virus RNA genome. J Virol 71: 8698-8706.

28. Taguwa S, Okamoto T, Abe T, Mori Y, Suzuki T, et al. (2008) Human butyrate-induced transcript 1 interacts with hepatitis C virus NS5A and regulates viral replication. J Virol 82: 2631-2641.

29. Ali N, Siddiqui A (1997) The La antigen binds 5' noncoding region of the hepatitis C virus RNA in the context of the initiator AUG codon and stimulates internal ribosome entry site-mediated translation. Proc Natl Acad Sci U S A 94: 2249-2254.

30. Liu HM, Aizaki H, Choi KS, Machida K, Ou JJ, et al. (2009) SYNCRIP (synaptotagmin-binding, cytoplasmic RNA-interacting protein) is a host factor involved in hepatitis C virus RNA replication. Virology 386: 249-256.

31. Gao L, Aizaki H, He JW, Lai MM (2004) Interactions between viral nonstructural proteins and host protein hVAP-33 mediate the formation of hepatitis C virus RNA replication complex on lipid raft. J Virol 78: 3480-3488.

32. Hamamoto I, Nishimura Y, Okamoto T, Aizaki H, Liu M, et al. (2005) Human VAP-B is involved in hepatitis C virus replication through interaction with NS5A and NS5B. J Virol 79: 13473-13482.

33. Backes P, Quinkert D, Reiss S, Binder M, Zayas M, et al. (2010) Role of annexin A2 in the production of infectious hepatitis C virus particles. J Virol 84: 5775-5789.

34. Benga WJ, Krieger SE, Dimitrova M, Zeisel MB, Parnot M, et al. (2010) Apolipoprotein E interacts with hepatitis C virus nonstructural protein 5A and determines assembly of infectious particles. Hepatology 51: 43-53.

35. Tellinghuisen TL, Foss KL, Treadaway J (2008) Regulation of hepatitis C virion production via phosphorylation of the NS5A protein. PLoS Pathog 4: e1000032.

36. Li Q, Krishnamurthy S, Lan KH, Chen W, Liang TJ. A novel NF-kB-independent function of IKKa in hepatitis C virus assembly; 2010 Sep. 10-14; Yokohama, Japan.

37. Menzel N, Fischl W, Hueging K, Bankwitz D, Frentzen A, et al. MAP-Kinase Regulated Cytosolic Phospholipase A2 Activity Is Essential for Production of Infectious Hepatitis C Virus Particles. PLoS Pathog 8: e1002829.

38. Herker E, Harris C, Hernandez C, Carpentier A, Kaehlcke K, et al. (2010) Efficient hepatitis C virus particle formation requires diacylglycerol acyltransferase-1. Nat Med 16: 1295-1298.

39. Zayas M, Romero-Brey I, Bartenschlager R. Role of bar proteins in production of infectious hepatitis C virus particles; 2010 Sep. 10-14; Yokohama, Japan.

40. Bishe B, Syed GH, Field SJ, Siddiqui A (2012) Role of Phosphatidylinositol 4-Phosphate (PI4P) and Its Binding Protein GOLPH3 in Hepatitis C Virus Secretion. J Biol Chem 287: 27637-27647.
